# Levetiracetam‐responsive paroxysmal exertional dyskinesia in a Welsh Terrier

**DOI:** 10.1111/jvim.16068

**Published:** 2021-02-27

**Authors:** Sherril Green, Natasha Olby

**Affiliations:** ^1^ Stanford University ‐ Comparative Medicine Stanford California USA; ^2^ North Carolina State University ‐ College of Veterinary Medicine Raleigh North Carolina USA

**Keywords:** movement disorder, paroxysmal dyskinesia, treatment, Welsh Terrier

## Abstract

A 5‐and‐a‐half‐year old, 9‐kg, spayed, female Welsh Terrier presented with a 12 month history of paroxysmal exertion‐induced dyskinesia (PED) characterized by recurrent episodes of involuntary hyperkinetic movements, abnormal muscle tone, and contractions triggered by exercise. A single episode occurred within 2 hours after exercise, lasted from 7 to 10 minutes, and resolved without treatment. The owner sought treatment for the dog when the episodes began to last longer (20‐30 minutes), and occurred as long as 2.5 to 8 hours after exercise. Diazepam administered intranasally at the start of an episode promptly alleviated the symptoms. Maintenance therapy with levetiracetam proved effective, such that the dog was gradually returned to exercise. However, attempts to wean the dog off the drug resulted in reoccurrence. Although the pathophysiology of PED is not fully understood, the clinical presentation and the positive response to antiepileptic therapy highlight the overlap between disease pathways in epilepsy and PED in dogs.

**Abbreviations**
AED: antiepileptic drug
CSF: cerebral spinal fluid
EEG: electroencephalography
MD: movement disorder
PD: paroxysmal dyskinesia
PED: paroxysmal exertion‐induced dyskinesia

## 1 INTRODUCTION

Paroxysmal dyskinesia (PD) refers to a group of movement disorders (MDs) characterized by recurring episodes of involuntary hyperkinetic movements, ballism, dystonia, athetosis, or chorea.^1^, ^2^, ^3^ The irregular muscle movements typically involve the limbs, trunk, neck and/or face, last minutes to several hours, and occur without a loss of consciousness. The PDs have been classified into 3 main types: paroxysmal kinesigenic dyskinesia (PKD), in which an episode is induced by an abrupt, voluntary physical movement; paroxysmal nonkinesigenic dyskinesia, in which episodes are not preceded by sudden movement or exercise; and paroxysmal exertion‐induced dyskinesia (PED), in which episodes are triggered by prolonged exercise.^4^ The phenomenology overlaps between the PDs and other types of MDs, and because diagnostic tests tend to show no specific abnormalities or are inconclusive, PD classification algorithms have been expanded to include additional triggers (eg, heat, cold, stress, anxiety, caffeine, alcohol, sleep, or waking from sleep), the age of onset, severity of clinical signs, episode frequency and duration, and the presence of coexisting signs (eg, ataxia, hemiplegia, migraines, parkinsonism, or epilepsy).^1^, ^5^ Paroxysmal dyskinesias as recognized in human patients might be associated with acquired conditions (eg, metabolic disturbances, toxicosis, or trauma), immune‐mediated disorders, vascular or neurodegenerative disorders, idiopathic, or attributed to known or suspected genetic mutations.^1^, ^2^, ^5^, ^6^, ^7^, ^8^, ^9^, ^10^, ^11^, ^12^, ^13^, ^14^, ^15^, ^16^, ^17^, ^18^, ^19^, ^20^, ^21^, ^22^, ^23^, ^24^

The PDs share phenomenology with, and can be difficult to distinguish from, focal epileptic seizures.^1^, ^2^, ^5^ Most PD patients have a positive response to antiepileptic medications, suggesting overlap between the disease pathways. However, consciousness is preserved in PD and electroencephalographic findings in PD patients are normal before, during, and after an episode. Thus, the PDs are considered distinct, nonepileptic disorders.

Movement disorders characterized as PD are increasingly reported in a number of dog breeds, including Cavalier King Charles Spaniels, Scottish Terriers, and Border Terriers, among others.^2^, ^17^, ^25^, ^26^, ^27^, ^28^, ^29^, ^30^, ^31^, ^32^, ^33^, ^34^, ^35^, ^36^, ^37^, ^38^, ^39^, ^40^, ^41^, ^42^, ^43^, ^44^, ^45^, ^46^, ^47^, ^48^, ^49^, ^50^, ^51^, ^52^, ^53^, ^54^, ^55^

Therapeutic management strategies for PD in dogs have relied on conventional antiepileptic drugs (AEDs; namely the benzodiazepines) alone, or in combination with carbonic anhydrase inhibitors, muscle relaxants, glucocorticoids and other anti‐inflammatory agents, or dietary changes. Levetiracetam, a novel AED which modulates neurotransmitter release by binding to the synaptic vesicle protein SV2A, has been used to treat epilepsy and PD since the early 2000s.^4^, ^5^, ^56^ Here, we report PED in a Welsh Terrier and long term management with levetiracetam.

### 1.1 Case history

A 5‐and‐half year old spayed, 9‐kg, female Welsh Terrier, presented with a 12‐month history of abnormal muscle contractions and “cramping” that occurred after prolonged exercise (within 15‐20 minutes after 30 minutes of ball chasing or after hour‐long hikes with the owner). The owner reported that initially, a single episode generally lasted from 7 to 10 minutes and resolved without treatment. The owner reported that the dog did not lose consciousness during an episode. The dog experienced cramping that started in a hind limb and progressed to involve all 4 limbs, and the back muscles, such that the dog assumed a stiffened, hunchbacked appearance.

The owner sought treatment for the dog when the episodes began to last longer (20‐30 minutes) and occurred repeatedly (2‐3 times) over an extended period (2‐8 hours) after exercise and were followed by emesis within 1 to 2 hours after the muscle contractions ceased. The dog became increasingly inappetent and lethargic and lost 1.4 kg of body weight over the previous 2 months. The owner ceased exercising the dog, but reported that the dog recently experienced an episode lasting ~20 minutes (Supporting Video S1 **)** that was triggered by a 15 minute walk before a trip to the veterinarians.

All vaccinations were current and the dog was maintained on milbemycin oxime (Trifexis, Elanco, Greenfield, Indiana) to manage external and internal parasites. The dog had been fed a balanced, grain free, gluten free, single source protein diet (Healthy Balance) for the last 3 years. Results from hematological and serum biochemical tests and urinalysis collected on 3 different occasions over the past 6 months, 2 of which had been collected within 20 minutes after an episode, did not reveal any abnormalities.

**FIGURE 1:**
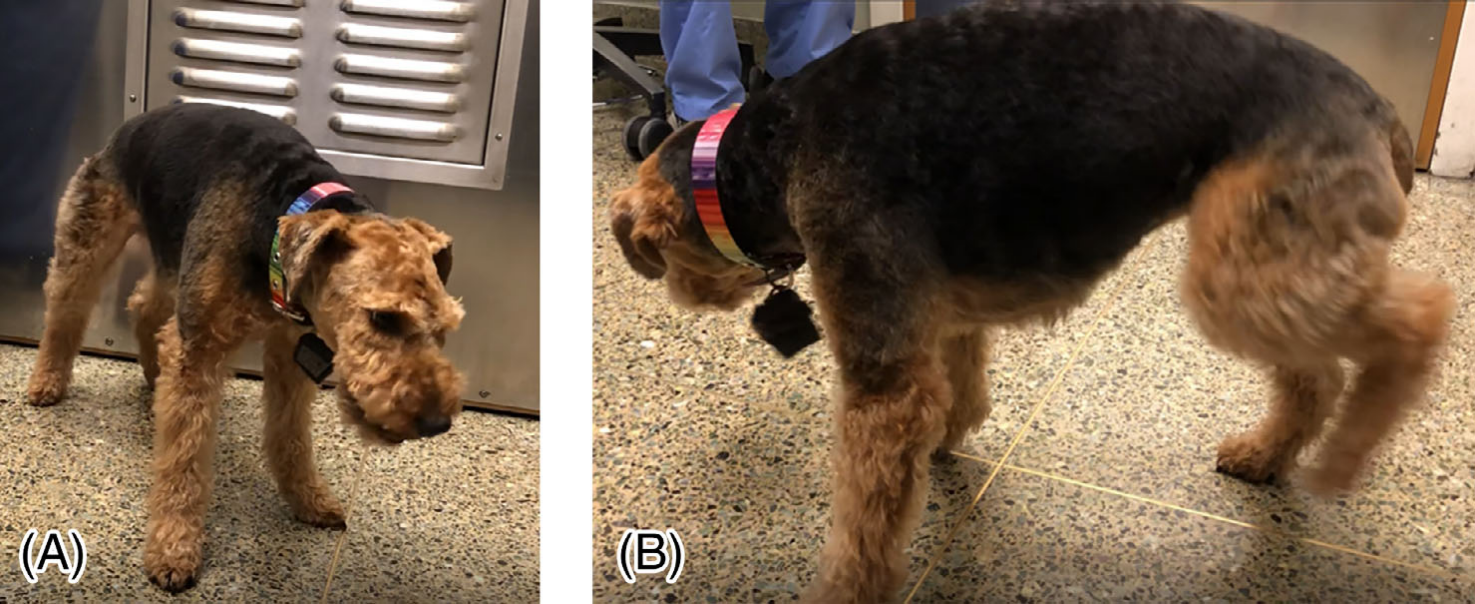
A 5‐and‐a‐half year old, 9‐kg, female Welsh Terrier with paroxysmal exertion‐induced dyskinesia (PED) showing an arched back due to lumbar and thoracic muscle contractions (A) and contraction of the hind limb (B)

Evaluation of the video‐taped episode revealed after exercise muscle tremors and fasciculation, contractions that usually began in a hind limb. Contractions progressed to involve the other limbs, the epaxial muscles, the trunk, and the neck. The dog assumed a stiffened posture with an arched back and the head in a lowered position. Photographs (Figure 1A,B) show the postural changes observed during the PED episode. During the episodes, the dog also panted, made lip‐smacking motions, and sought comfort from the owners. The dog remained alert and responsive to the owners' commands, and did not show altered mentation, or blindness. The episodes were not characterized by a prodrome, ictal, or post ictal behavior. The dog often lay down, but did not collapse or fall over. She remained recumbent until the large‐muscle contractions and most of the fine muscle tremors and fasciculation rescinded. Upon cessation of an episode, the dog quickly fully recovered and ambulated normally.

The initial differential diagnoses included Canine Epileptoid Cramping Syndrome, previously known as Spike's disease, as reported in Border Terriers, or a condition similar to Scottie Cramp as seen in Scottish Terriers, or similar to the well‐recognized PD known as episodic falling syndrome as described in King Charles Cavalier Spaniels. Epilepsy in dogs was also considered as a differential diagnosis. Upon review of the videos, the dog did not appear to lose consciousness during the episodes and responded to the owners' commands. Given that dystonic episodes were consistently triggered by prolonged exercise and occurred without a loss of consciousness, PED was considered as the working diagnoses.

A treatment regimen was implemented with the goal of mitigating the frequency and duration of the episodes, such that the dog's quality of life could be improved. Therapeutic agents which were safer and with fewer adverse effects were empirically selected. Exercise remained restricted and the dog was initially placed on the short acting muscle relaxant methocarbamol (Robaxin, Endo Pharmaceuticals, Malvern, Pennsylvania; starting dosage, 250 mg PO, BID for 2 weeks), and the antiepileptic gabapentin (Neurontin Pfizer, New York, New York; starting dosage, 250 mg PO, BID). The owner slowly reintroduced light exercise (15 minute walks) over the following month and administered 2 mg intranasal diazepam (Diazepam, Hospira Inc, Lake Forest, Illinois), in the event an episode occurred. An antiemetic, maropitant citrate (Cerenia, Zoetis, Kalamazoo, Michigan; starting dosage, 24 mg PO, SID) was also administered to ease after episode vomiting. During this initial 4 week treatment period, the owner observed that treatment with methocarbamol and gabapentin did not prevent the episodes after light exercise. Intranasal diazepam, however, was very effective at stopping the episodes, usually within a minute or less after administration. After treatment with diazepam, the dog was notably sedated and somewhat lethargic. The owner observed that it appeared to be important to interrupt the progression of the episode by immediately administering intranasal diazepam at the onset. If left untreated, the episodes tended to last longer and increase in severity. Because methocarbamol and gabapentin were ineffective, the owner discontinued both medications and placed the dog on levetiracetam (Keppra, UCB, Smyrna, Georgia; 250 mg, PO, TID). After 2 weeks of treatment with levetiracetam, the owner did not observe any episodes, even after moderate exercise (20‐30 minute walks). Subsequent genetic testing for mutations in the BCAN^52^, ^53^ and *PIGN* ^55^ gene was performed and was negative.

After 8 months of treatment, the owner attempted to lower the dosage of levetiracetam with the intent to eventually wean the dog off of the medication. Three attempts, 2 weeks apart, to lower the dosage to 125 mg, PO, q8h, resulted in reoccurrence of the PD after light exercise, although the episodes were very mild and short in duration. The dog has since remained on treatment with levetiracetam, 250 mg, PO, q8h, given light exercise (30‐60 minutes of walking/day) and allowed short (<10 minutes), infrequent sessions of ball chasing and has had only 1 mild episode in the past 47 months.

## 2 DISCUSSION

This is a report of PED in a Welsh Terrier that describes a successful therapeutic approach to the long‐term management using the novel AED levetiracetam. Although the findings reported here must be interpreted within the limitations of a single case, the incidence of PED in Welsh Terriers is currently unknown. However, it is difficult to diagnose and therefore might be underreported. It is unknown if other members of this Welsh Terrier's pedigree are affected.

The phenomenology in this Welsh Terrier overlaps with PD disorders in other dog breeds. Paroxysmal dyskinesia in Border Terriers is also characterized by recurrent episodes of abnormal involuntary muscle movements, with no loss of consciousness.^31^, ^32^, ^57^ However, the episodes were not preceded by prolonged exercise, and were triggered by waking from sleep or by excitement. Approximately ~20% to 30% of affected Border Terriers display autonomic signs (borborygmi, urination, and diarrhea).^57^ Various treatments had variable‐to‐good outcomes over the short term, including the administration of conventional AEDs.^57^ Switching affected dogs to a gluten‐free diet, in conjunction with pharmaceutical therapeutics, reportedly ameliorated the clinical signs.^58^ Although the Welsh Terrier described here displayed similar PD signs, a consistent trigger for this dog was prolonged exercise. The dog in this report sometimes experienced emesis after episode, but other autonomic signs were not present. The dog in this report had been on gluten‐free diet for 3 years prior to the diagnosis and had experienced PED during that time, suggesting that a gluten‐free diet did not have an impact on the condition. Notably, switching to a ketogenic diet ameliorates the symptoms in some human patients with PD.^1^ This effect may be related to an alternate energy source in the food, rather than antiepileptic properties of the diet, per se. Feeding a ketogenic diet was not attempted in the dog described in this report.

Comparatively, the PD phenomenology in this Welsh Terrier shares more overlapping features with those seen in Scottish Terriers^17^, ^34^, ^35^, ^36^, ^40^, ^44^, ^59^, ^60^ with Scottie Cramp and with those as reported in King Cavalier Charles Spaniels^28^, ^30^, ^42^, ^46^, ^50^ with PD, including: exercise as a trigger, episodes that involved increasing muscle tone and stiffness of the extremities, postural arching of the back, no loss of consciousness, and responsiveness to treatment with conventional antiepileptic medications. The Welsh Terrier described here did not however, collapse or fall over, or assume a “praying position” during a PD episode (as described in King Cavalier Charles Spaniels) or fall or summersault when running, or collapse during exercise and recover in a few minutes, only to collapse and recover again after continuing to exercise as observed in Scottish Terriers with Scottie Cramp.^44^, ^59^

Epilepsy in dogs was also considered as a differential diagnosis in the dog described in this report, but distinguishing PD in dogs from an epileptic disorder poses a particular diagnostic challenge. An increased ratio of cerebral spinal fluid (CSF)/serum glucose is a useful diagnostic tool in human PED, and the electroencephalography (EEG) indices in human medicine are helpful in ruling out seizure disorders, but CSF/serum glucose ratios in PDs in dogs are not described and EEG indices in epilepsy in dogs are not as well characterized.^60^, ^61^, ^62^ Assessing loss of consciousness or impairment of consciousness in epilepsy of dogs is an added challenge, as the EEG indices in dogs have not been well‐established.^62^ Conscious impairment in dogs is generally assessed subjectively, by evaluating the dog's ability to respond to owners' commands.^49^ The Welsh Terrier described here does not appear to lose consciousness or experience conscious impairment. She readily responds to the owners' command to “come here.” Other tests that have the potential to clarify the ante‐mortem diagnosis, such as muscle biopsies, computerized tomography, or magnetic resonance imaging of the brain can be cost prohibitive or impractical in veterinary medicine and might not provide additional diagnostic information regarding the PDs.^1^, ^5^, ^60^, ^61^ Although this dog tested negatively for mutations for the BCAN and *PIGN* gene, mutations reported to cause PD in Cavalier King Charles Spaniels and Soft‐Coated Wheaten Terriers, respectively, tests developed for 1 breed may produce falsely negative results if the new breed has a different mutation.

Antiepileptic drugs as first‐line therapeutics for the PD, especially for the PKDs, are reported to be effective in human patients and are often given in combination with other medications.^5^ The muscle relaxant methocarbamol and the AED gabapentin, both of which are considered relatively safe and have minimal adverse effects, did not ameliorate the PED in this Welsh Terrier. However, diazepam, a benzodiazepine and GABA agonist, when administered intranasally immediately at the start of an episode, effectively halted the dog's PD. Levetiracetam alone was highly effective in controlling this dog's PED over the long term, such that the dog was able to return to exercise. There are reports that PD might be self‐limiting in humans and in dogs.^49^, ^53^, ^58^ However, attempts to wean the dog described in this report off of the medication resulted in reoccurrence of disease.

The use of levetiracetam for the treatment of epilepsy and of PD in humans is well documented.^56^ It offers the pharmacokinetic advantages of rapid and near complete absorption when given orally, the absence of interactions with other drugs, the absence of enzyme induction and insignificant binding to plasma protein, among others.^56^ Levetiracetam was efficacious in the treatment of PED in this dog, and there were no long‐term adverse effects after 47 months. It remains unclear how levetiracetam acts specifically to control or ameliorate the symptoms of PD, although modulation of the synaptic neurotransmitter release in the brain probably plays a role. The animal's response to these 2 AEDs, diazepam in the short term and levetiracetam thereafter, further supports the tenet that epilepsy and PD share pathophysiological pathways.

## CONFLICT OF INTEREST DECLARATION

Authors declare no conflict of interest.

## OFF‐LABEL ANTIMICROBIAL DECLARATION

Authors declare no off‐label use of antimicrobials.

## INSTITUTIONAL ANIMAL CARE AND USE COMMITTEE (IACUC) OR OTHER APPROVAL DECLARATION

Authors declare no IACUC or other approval was needed.

## HUMAN ETHICS APPROVAL DECLARATION

Authors declare human ethics approval was not needed for this study.

## Supporting information


**Video S1** A five‐and‐a‐half year old Welsh Terrier experiencing PED after exercise. Note the arched back, panting, contraction of the hind limb and low head and neck carriage. This PED episode lasted approximately 20 mins. Video was taken with an iPhone 10 and formatted with Adobe Premiere (San Jose, CA).
